# Performance Under Pressure in Sport: A Position Statement from Exercise and Sports Science Australia (ESSA)

**DOI:** 10.1007/s40279-026-02472-x

**Published:** 2026-06-10

**Authors:** Matthew J. Schweickle, Renee N. Appaneal, Patricia C. Jackman, Clare L. Minahan, Gene M. Moyle, Craig Pickering, Damien Stewart, Stewart A. Vella, Christian Swann

**Affiliations:** 1 Global Alliance for Mental Health and Sport, School of Psychology, Faculty of Science, Medicine and Health, University of Wollongong, Wollongong, NSW 2500 Australia; 2 Prepare and Perform, Canberra, ACT Australia; 3 School of Psychology, Sport Science, and Wellbeing, University of Lincoln, Lincoln, UK; 4 Griffith Sports Science, Griffith University, Gold Coast, QLD Australia; 5 Female Performance and Health Initiative, Australian Institute of Sport, Canberra, ACT Australia; 6 Faculty of Creative Industries, Education and Social Justice, Queensland University of Technology, Brisbane, QLD Australia; 7 Australian Athletics, Melbourne, VIC Australia; 8 Room23 Psychology, Brisbane, QLD Australia; 9 Movember, Melbourne, VIC Australia; 10 Physical Activity, Sport and Exercise Research Theme, Faculty of Health, Southern Cross University, Coffs Harbour, NSW Australia

## Abstract

Supporting performance under pressure is important to many roles in the sporting sector, including Exercise and Sports Science Australia (ESSA) Accredited Sport Scientists and Accredited High Performance Managers. This position statement on behalf of ESSA aims to support practitioners whose responsibilities include facilitating athletes’ performance under pressure. To achieve this aim, we synthesised relevant systematic reviews, and theoretical and empirical publications, regarding supporting performance under pressure in sport. Specifically, we focused on translating the scientific literature regarding the different sources of pressure athletes may experience, how athletes may respond to this pressure, the different strategies that may be used to support performance under pressure and considerations for how performance under pressure may be evaluated. This position statement highlights that pressure can have both facilitative and debilitative effects on athlete performance. Training environments offering implicit learning opportunities and effective utilisation of pressure training can prepare athletes for performing well under pressure. A range of evidence-based psychological strategies can be utilised when performing under pressure, including: (1) pre-performance routines, (2) implicit attentional strategies, (3) psychological skills training and (4) mindfulness-based approaches. Suggestions are provided for how to appropriately select and implement different strategies depending on sport type or context. Drawing on a systems-led approach, performance teams can collectively work together to support athlete performance under pressure in an integrated manner. This position statement concludes with eight overarching recommendations to support performance under pressure. These recommendations are contextually specific to key stakeholder groups, such as the organisation, performance team members and sports researchers.

## Key Points

**Table:** 

All members of a performance team play a role in supporting athletes’ performance under pressure.
Training environments offering implicit learning opportunities and effective utilisation of pressure training can prepare athletes for performing well under pressure.
Individual psychological strategies, such as pre-performance routines, implicit attentional strategies, psychological skills training and mindfulness-based approaches, can all support performance under pressure.

## Introduction

Performing under pressure is a fundamental aspect of sport [1]. Supporting performance under pressure is important to many roles in the sporting sector, including Exercise and Sports Science Australia (ESSA) Accredited Sport Scientists and Accredited High Performance Managers. Accordingly, the intended audience of this position statement are practitioners whose role includes supporting athletes’ performance under pressure, who we refer to throughout as the performance team.

This position statement draws on a systems-led approach [2]. Such an approach recognises that sport organisations contain multiple stakeholder groups with diverse responsibilities and dynamics, yet which are interconnected and complementary. In the context of performance under pressure, multiple stakeholders will inevitably play important roles and hold different responsibilities in supporting performance under pressure. The purpose of this position statement is to synthesise and translate core concepts and theories regarding performance under pressure—a field that has typically been approached through a psychological lens—to extend all relevant members of the performance teams’ knowledge and understanding of performance under pressure. This knowledge can contribute to the development of more psychologically informed environments [2], in which psychological concepts, such as pressure, are more widely understood, discussed and owned by a range of stakeholders in the organisation.

Specifically, we aim to: (1) outline key sources of pressure within sport and how pressure relates to overlapping constructs, (2) describe potential performance responses to pressure and the theoretical explanations underpinning these performance responses, (3) discuss how psychologically informed training environments can be designed to support performance under pressure, (4) review psychological strategies that can be used to support performance under pressure and their implementation and (5) outline considerations for evaluating performance under pressure. To inform this position statement, we drew on systematic reviews related to performance under pressure [3–11] and key psychological strategies [12–22] in sports, relevant theories (e.g. [23–26]), empirical studies (e.g. [27–29]) and applied recommendations for supporting performance under pressure (e.g. [30–32]). The synthesis was guided by the aims of the position statement, with a specific focus on identifying practical recommendations that were relevant to the target audience and in line with robust theory and evidence. We conclude with a series of recommendations that are contextually specific to key stakeholder groups, such as the organisation, performance team members and researchers. These recommendations are signposted throughout the position statement to highlight the context in which they have arisen.

## What is Pressure in Sport, and What Influences it?

Pressure is widely defined (e.g. [4, 6–9, 27, 30, 31]) using Baumeister’s (1984) definition of ‘any factor or combination of factors that increases the importance of performing well on a particular occasion’ [33]. A range of environmental, subjective and task-related factors have been demonstrated to influence the experience of pressure in sport. Recognised sources of pressure include rewards or punishments [27, 34–40], presence of an evaluative audience or competitors [27, 34, 36–44], limited chances or reduced time to complete a task [27, 34], increased task difficulty [27, 40], previous performance errors [43, 45–47], internal expectations and goals [38, 42, 48, 49] and perceived external expectations (e.g. parents, coaches [38, 42, 48, 50]). These sources of pressure are not equal or additive [27], in that athletes may respond differently to the same factor, whilst the presence of such factors does not mean an athlete will always experience pressure [46, 48]. For the experience of pressure to occur, the athlete must both be aware of the incentives to perform well and be motivated to perform well in response to these incentives [34]. Further, such awareness and perception of importance may fluctuate before and during an event [46, 48]. In sum, pressure represents a distinct psychological episode related to a performance, in which the athlete is both aware of the importance of performing well, and motivated do so.

Performance team members may also experience pressure in relation to their own performance expectations, as well as broader workplace stressors [51, 52]. For performance team members, these may include challenging interpersonal relationships with colleagues or athletes, and excessive expectations regarding job responsibilities, availability and performance [51]. Such pressures can have a downstream effect on athletes. For example, Thelwell et al. [53] reported that athletes often perceive when a coach is experiencing stress or pressure, which in turn, may increase the athlete's own perception of pressure. Similarly, Lachini [54] reported that external pressures on coaches can result in less autonomy-supportive coaching behaviours, which may in turn increase pressures on athletes (e.g. [55]). Performance team members, therefore, may also consider utilising relevant strategies within this statement to help manage performance pressures (e.g. [52]), whilst organisations should aim to play an active role in supporting and managing stressors placed upon performance team members ([51, 56]; see Recommendation 3, Fig. 2).

### Stress and Performance Anxiety

Whilst the concepts of pressure, stress and performance anxiety are often used interchangeably (e.g. [9]), these are distinct concepts. Stress refers to an ‘an ongoing process that involves individuals transacting with their environments, making appraisals of the situations they find themselves in, and endeavouring to cope with any issues that may arise’ [57]. Stress may occur in response to a stressor, which are environmental demands encountered by an individual [57]. Of note, stressors may relate to performance, but also to the preservation of one's wellbeing [58–60]. For example, stressors may be organisational (e.g. travel for a competition), competitive (e.g. underperformance), interpersonal (e.g. strained relationship with a coach) or personal (i.e. non-sporting related), and may be acute (e.g. a decision from the umpire) or chronic (e.g. extended injury rehabilitations [61]). Beyond these categorisations, the characteristics of a stressor may influence one's physiological response. For example, in a meta-analysis examining non-sport performance tasks, Dickerson and Kemeny reported that stressors characterised by uncontrollability and/or social evaluative threats produced greater cortisol responses than stressors without these characteristics [62]. Whilst stressors may be broad in both their source and their characteristics, stressors that increase the importance of performing well on a particular occasion are considered a source of pressure [33, 61].

Whether an athlete experiences stress in response to a stressor is a product of appraising, defined as ‘the evaluative process by which stressors are evaluated and relational meanings constructed’ [63]. Such appraising involves determining if the stressor is significant to one’s values or goals (i.e. primary appraisal) and whether one perceives sufficient resources to cope with the stressor (i.e. secondary appraisal [64]). In the context of performance, such resources may include one's perceived psychological attributes (e.g. self-efficacy [65]), social support (e.g. team, coaches, performance team [66]) and learned performance strategies, for example. When an athlete appraises a discrepancy between a stressor and their ability to cope, performance anxiety may occur. Performance anxiety has been defined as ‘an unpleasant psychological state in reaction to perceived threat concerning the performance of a task under pressure’ [67], and involves both cognitive (i.e. worry and self-focus) and somatic (i.e. autonomous hyperactivity and somatic tension) components [67]. Whilst it is often assumed that pressure leads to increased performance anxiety (e.g. [35]), this relationship largely depends on how one appraises their own resources in response to pressure [24]. Further, the experience of performance anxiety does not always result in poor performance. For example, the Theory of Challenge and Threat States [68] highlights how athletes can facilitatively interpret symptoms of performance anxiety, particularly when demonstrating high levels of perceived control (e.g. [69]) and self-efficacy (e.g. [70]), and when setting approach-orientated goals (see Sect. 5.3), and experience challenge states whilst performing under pressure. Accordingly, strategies to support performance under pressure aim to provide a dual function of supporting athletes’ initial appraisal of their resources to respond under pressure, whilst also supporting the ability to perform with anxiety if or when it does occur.

Whilst one’s resources play a critical role in how pressure is appraised and whether performance anxiety occurs, personal traits and sociocultural factors are also influential. For example, personal traits such as sensitivity to rewards and punishment [71], narcissism [72, 73], public self-consciousness [39], self-presentation concerns [38] and dispositional reinvestment [74] may all influence how an individual responds under pressure. Further, women athletes and young athletes have reported higher levels of overall trait anxiety [75], whilst para-athletes have reported experiencing a range of sport- and impairment-specific stressors that do not commonly affect athletes without disabilities [76, 77]. Athletes who experience stereotype threat, meanwhile, which refers to the societal linking of a particular group of people to a specific performance outcome or trait [78], may experience increased performance anxiety in pressurised settings [79]. Whilst such stereotypes are both culturally- and sport-specific, they may be more likely to negatively impact women athletes, athletes of varied cultural backgrounds or ethnicities, para-athletes and older athletes [80]. These examples are not exhaustive, but they highlight the importance of considering how both personal traits and the sociocultural environment of sport may impact athletes' experience of pressure and performance anxiety.

## Performance Responses to Pressure in Sport

Athletes may demonstrate debilitative or facilitative performance responses under pressure. 'Choking' under pressure reflects an acute and considerable decrease in performance because of increased performance anxiety and lack of perceived control in response to pressure [81]. In contrast, 'clutch' performance represents a facilitative performance response characterised by high levels of perceived control and deliberate task-focused attention [49]. Whilst clutch performance was initially defined as a superior performance under pressure circumstances [41], recent qualitative studies with athletes suggest that clutch performance specifically refers to when an athlete achieves their performance-related goals under increased pressure [48, 82].

Theoretical models that explain the role of pressure on performance outcomes have largely focused on the critical role of task-relevant attention [3, 4, 23]. These theoretical models posit that in response to increased performance anxiety, athletes’ attention may be diverted away from the task at hand, resulting in performance breakdown. The two broad categorisations of attentional theories of choking are self-focus theories (e.g. explicit monitoring hypothesis, reinvestment theory [26, 83]) and distraction theories (e.g. attentional control theory [24, 25]). Self-focus theories specify that in response to increased performance anxiety, attention is directed towards consciously monitoring and controlling skill execution, which reduces automaticity and overall performance [26]. Distraction theories posit that in response to increased performance anxiety, athletes’ attention is diverted towards external (e.g. crowd) or internal (e.g. worries) task-irrelevant stimuli [24]. Interventions informed by attentional theories draw upon a range of strategies to either correct attentional disruption or maintain attentional focus. Accordingly, the maintenance of task-relevant attention is considered critical to performance under pressure.

## Creating Training Environments to Support Performance Under Pressure

The content and design of the training environment may support an athlete's preparation to perform under pressure in competition. Teaching athlete's skills in an implicit manner, where skills are acquired without learning explicit step-by-step instructions, can support performance under pressure. Specifically, through bypassing the declarative knowledge stage, it is suggested that skills are less likely to breakdown under reinvestment [26] and are more likely to be performed with automaticity [84]. For example, a practitioner may utilise analogy-based instructions to support implicit learning [85]. Dual-task conditions, hemispheric priming, and quiet eye training (see Sect. 5.2 for an overview of these strategies) may also support implicit learning. In a meta-analysis of 10 studies, Cabral and colleagues [7] reported a medium positive effect of implicit learning on performance under pressure. The studies reviewed, however, primarily contained novice athletes learning a new skill, potentially limiting the utility for more well-trained athletes. In sum, practitioners can utilise implicit learning approaches, particularly with those developing skills (e.g. youth athletes in high-performance pathways [86]), when aiming to prevent future skill breakdown under pressure.

Training environments should be designed to reflect the physical and psychological demands of competition. Representative learning designs prioritise both fidelity (i.e. closely matching the physical skills to those required in competition [87]) and functionality (i.e. exposing athletes to comparable, yet controlled, sources of perceptual information experienced in competition [87]) and may support athletes’ performance in pressurised environments. Developing core cognitive skills that facilitate performance under pressure—such as attention—may play an important role in creating a representative training environment (e.g. [88]). Consideration should also be given to the extent to which the training environment reflects the emotional demands of competition (i.e. affective learning design [89]), particularly when considering the role of emotions (e.g. performance anxiety) in performing effectively under pressure.

Pressure training refers to the manipulation of the practice environment with the intent to create a stress-related response [5, 30, 31]. In doing so, athletes are provided with the opportunity to develop or practice coping strategies, gain greater awareness into their own cognitions, emotions, and behaviours when under pressure, and ultimately, increase their familiarisation with pressure situations [30]. A recent meta-analysis demonstrated that pressure training had a moderate effect on supporting performance under pressure [5]. In line with representative learning design principles (e.g. [87]), the manipulation of pressure within the training environment should be as context specific as possible yet may draw on examples such as simulating competitive scenarios, introducing rewards, forfeits/consequences, or distractions, or limiting the number of chances to complete the task [10, 31].

Pressure training must be appropriately implemented to enhance performance and avoid potential risks to athlete wellbeing. Excessively imposed pressure without adequate support may lead to an unrelenting sport culture [90] or cause strain between the coach and athlete [91]. Given the potential for such adverse effects, pressure training should be delivered in close collaboration with an accredited sport and exercise psychology practitioner [92]. To support the effective implementation of pressure training, performance teams should: (1) provide the opportunity to develop strategies to support performance under pressure prior to the introduction of pressure training; (2) gradually introduce the amount of pressure by manipulating common sources of pressure; (3) avoid engaging in pressure training too often or during the learning of new skills; (4) communicate the rationale for pressure training to athletes and encourage athlete 'buy-in'; (5) monitor, and if necessary modify, pressure training; and (6) debrief following the implementation of pressure training and evaluate whether the desired outcomes were achieved (for detailed descriptions of these processes see [10, 30, 31, 93]; Recommendation 4, Fig. 2).

## Psychological Strategies to Facilitate Performance Under Pressure

As part of fostering a systems-led approach to supporting performance under pressure, in which the whole performance team plays a role in supporting performance under pressure (see Recommendation 1, Fig. 2), it is important for key stakeholders to understand the different reasons why certain psychological strategies may be applied. This shared understanding can enable consistent communication regarding performance under pressure across all levels of the sports organisation, from the performance team to athletes. One approach which has had a large influence within the field is to utilise cognitive-behavioural strategies to support an athlete's ability to control or change internal experiences (i.e. second-wave cognitive-behavioural approaches). Specifically, this may involve a focus on regulating unpleasant emotions such as performance anxiety and challenging the validity of the thoughts underlying this emotion [94]. By contrast, other approaches—largely stemming from Acceptance and Commitment Therapy [95]—encourage athletes to utilise strategies intended to enable them to accept and make room for (rather than trying to control or avoid) unpleasant thoughts and feelings that arise in pressured contexts, whilst remaining engaged with the task and committing to values-guided behaviours (i.e. third-wave cognitive behavioural approaches [32, 96]). As demonstrated below, both approaches may be effective in supporting performance under pressure, with the decision of which approach to use shaped by the athletes’ goals and circumstances, the practitioners’ therapeutic orientation and consulting philosophy [97, 98], and the organisational and cultural context [99]. To most effectively support athletes in a unified manner, performance team members should ensure alignment in how they communicate with athletes to support performance under pressure.

Both the psychological strategies presented below, and the considerations for creating psychologically informed training environments presented above, should be applied in a critical and contextually appropriate way (see Recommendation 5, Fig. 2). Given that the same strategy may be utilised for different purposes (e.g. breathing exercises may be used for both arousal management or to encourage mindful focus; 105]), it is important the performance team is aware of the intended outcome of strategies and ensure these outcomes align with the approach adopted. The effectiveness of such strategies may also be influenced by an athlete's individual needs and preferences, and the working relationship between an athlete and practitioner [100, 101]. Accordingly, we recommend that where feasible, these strategies are supported by an accredited sport and exercise psychology practitioner or an individual on a recognised accreditation pathway (see Recommendation 2, Fig. 2). Within the context of Australia this is a registered psychologist, ideally with endorsement, or the eligibility to be endorsed, in sport and exercise psychology [102]. Of note, credentialing systems and accreditation of practitioners differ between nations and regions (see [103]), and for practitioners working in other countries, titles may differ (see Table 1). Whilst such support may involve working directly with athletes, this support can also extend to contributing towards building more psychologically informed environments across the performance team and organisation. That is, shared models of practice can be fostered where all members of the performance team work together based on principles of participation, shared responsibility, and mutual learning to support athlete performance under pressure [104]. Such models of practice aim to avoid silos or misalignment across roles and may be facilitated by regular multidisciplinary team meetings and having a specific role responsible for oversight of the performance environment (see [2] for full discussion). Presented below in Table 1 are key considerations when selecting potential strategies to support performance under pressure, as well as examples of how these considerations may be applied.

**Table 1:** Considerations for selecting strategies to support performance under pressure

Consideration	Rationale	Application
Is this strategy applicable to the sport and context?	Strategies to support performance under pressure may be most beneficial when they can feasibly be implemented in the relevant sport and context. Not all strategies are appropriate for all contexts and adaptations may need to be made to enable implementation in some contexts	Pressure training: appropriate across all sports where the athlete or team is likely to experience performance pressure, and where the athlete/team goals are performance orientated Pre-performance routines: most appropriate prior to self-paced, closed skills in individual (e.g. golf, tennis serve, gymnastics) or team (e.g. water polo penalty shot, basketball free throw) sports Implicit attentional strategies: most appropriate prior to (i.e. hemisphere-specific priming, quiet eye training) or during (i.e. dual-task strategies) self-paced, closed skills in individual (e.g. gymnastics, golf) or team (e.g. soccer penalty shot) sports Psychological skills training: applicable to all sporting contexts Mindfulness-based approaches: applicable to all sporting contexts
Can the teaching and development of these strategies be supported in practice?	Strategies to facilitate performance under pressure may be most effective when their teaching, use and development are supported cohesively by all members of the performance team. Accredited sport and exercise psychology practitioners may work with both athletes and other members of the performance team to support the development and implementation of these strategies	Whilst titles and registration pathways differ between nations and regions, examples include: Australia: Registered psychologist (ideally with, or eligible for, endorsement in sport and exercise psychology; Psychology Board of Australia) USA: Certified mental performance consultant (Association for Applied Sport Psychology) United Kingdom: Sport and exercise psychologist/practitioner psychologist (Health and Care Professions Council)
Does the use of strategies align with how the relationship between pressure and performance is viewed?	Where one aims to reduce or control the experience of pressure and performance anxiety, strategies which align with this approach may be most beneficial. Where one aims to accept and make room for the experience of pressure and performance anxiety, strategies which align with this approach may be most beneficial	Whilst strategies can be adapted to different paradigms (e.g. [105]), typically, psychological skills training and implicit attentional strategies may be viewed as reduction or control focused, whilst mindfulness-based approaches may be viewed as acceptance-focused
Will strategies be combined?	Individual strategies may be utilised; however, strategies can also be combined. When combining strategies, consideration should be given to the alignment between control versus acceptance-based approaches	Pre-performance routines may involve other strategies (e.g. self-talk, imagery, arousal management, implicit attentional strategies, mindfulness) Psychological skills training may be combined with implicit attentional strategies Psychological skills training may be integrated with mindfulness-based approaches, if content and focus are aligned (see [105])
Is there an opportunity for athletes to contribute to the choice and development of strategies?	Providing an athlete the opportunity to contribute to the choice and development of strategies they are to use is likely to support athlete buy-in, motivation and implementation	Through both individual and team-based consultation, a person-centred approach can be adopted in which athletes are provided with the choice of the strategies they use and how these are implemented [100, 106]. Providing choice can sit under a unified approach shared amongst the team and organisation e.g. [96], which focuses on developing a culture where athletes are both provided with a range of options to try and are supported in discovering what, or in what way, different strategies work best for them
Are there opportunities to practice the strategies?	Before implementation in competitive settings, it is beneficial to provide athletes with the opportunity to practice strategies in non-pressurised settings. Regular practice of strategies will also support their effectiveness	Athletes should have the opportunity to practice strategies in non-competitive and learning-focused settings, before using such strategies in competition or during implementation of pressure training

### Pre-performance Routines

Pre-performance routines (PPR) refer to a set of task-relevant thoughts and actions an athlete systematically engages in prior to task execution [8]. PPRs differ from superstitious or ritualistic behaviours, which are typically less intentional and attribute an external locus of control [107], and in some instances, can involve rigid behaviours which may compromise athlete wellbeing [108]. A recent systematic review of PPRs in sport demonstrated that utilising a PPR facilitated performance under pressure regardless of participant age, gender, or skill level or whether the PPR consisted of a single element (i.e. a stand-alone PPR) or combined multiple elements (i.e. an extensive PPR) [8]. For example, in a bowling task, Mesagno and colleagues [109] reported that PPRs which consisted of a single element (i.e. hemisphere-specific priming), multiple individualised elements (e.g. deep breaths, focusing on target, cue words) or a combined PPR (i.e. multiple elements combined with hemisphere-specific priming) were all effective at improving performance under pressure against a control group, with no differences reported in performance between the different types of PPRs. Further, athlete buy-in and motivation may be heightened when athletes are involved in the construction of the PPR [8]. Of note, PPRs have typically been examined in the context of self-paced, closed skilled environments. Several qualitative studies [28, 38], however, have highlighted that broader pre-performance preparation (e.g. developing proactive coping strategies for something going wrong during the performance) was perceived to support performance under pressure in more open skilled environments. PPRs, therefore, provide a method through which many of the strategies described below can be incorporated into an athlete's preparation to support performance under pressure.

### Implicit Attentional Strategies

Several strategies have been suggested to facilitate performance under pressure, which centre around deliberate efforts to direct visual or mental attention away from skill execution [12]. These strategies include dual-task conditions, hemisphere-specific priming and quiet-eye training. Dual-task strategies involve using a secondary cognitive task to draw attention away from conscious motor processing (e.g. counting backward from 100 by twos, listening to music [110, 111]). In a golf-putting experimental task, Land and Tenenbaum [112] reported that skilled participants who engaged in a dual-task condition performed better under pressure than a control group. Hemisphere-specific priming involves contracting one's left-hand (e.g. by squeezing a soft ball), twice a second, for 30–45 s prior to task execution. Gröpel and Beckmann [113] reported that gymnasts who engaged in left-hand contractions during a university championship final performed better than a comparison group. Given the use of additional stimuli in hemisphere-specific priming (e.g. squeezing a soft ball) and variations of dual-task strategies (i.e. listening to music), consideration should be given to whether the use of such stimuli is feasible within competitive environments. Lastly, quiet-eye training, which refers to efforts to support the visual fixation of a task-relevant target prior to skill execution, has demonstrated performance benefits. Wood and Wilson [114] showed that university-level soccer players who engaged in quiet-eye training (i.e. instructed to fixate on the preferred top corner of the goal) prior to a penalty shooting task under pressure performed better than a control group. Whilst such attentional strategies have broadly demonstrated encouraging results for performance under pressure (see [6]), they are largely only applicable to certain types of tasks (i.e. self-paced, discrete skills) and may not be applicable in more open skilled or externally paced environments.

### Psychological Skills Training

The most common strategies targeted in psychological skills training (PST) are goal setting, imagery, self-talk, and arousal management [115], and are used to enhance a range of psychological processes (i.e. self-efficacy, motivation), including task-focused attention.

Goal setting refers to the process through which people establish aims or objectives for their actions [116]. Goal setting may be considered in terms of goal type, goal orientation, and goal specificity. Regarding goal type, a recent systematic review and meta-analysis [13] demonstrated that process goals (i.e. the execution of behaviours, strategies, or thought processes [117]) and performance goals (i.e. self-referenced goals centred on the end product of performance [117]) significantly increased performance and attentional focus, and reduced performance anxiety. In a series of experiments examining a range of tasks (i.e. long jump, basketball shooting, golf putting), Mullen and Hardy [118] reported that holistic process goals (e.g. ‘drive’) were more effective for performance under pressure than part process goals (e.g. ‘arch back’), potentially owing to the reduced likelihood of conscious processing when utilising a holistic process goal. Regarding goal orientation, setting goals where the aim is to strive for competence (i.e. approach goals), as opposed to goals where the aim is to avoid incompetence (i.e. avoidance goals) may support challenge appraisals under pressure [119]. Lastly, Williamson and colleagues [120] reported that elite athletes perceived both specific and non-specific goals as effective for performance. Non-specific goals were reported to reduce perceptions of pressure, anxiety, and stress, whilst specific goals supported motivation. Swann and colleagues [49] reported that athletes who performed optimally under pressure set specific goals, typically towards the end of an event. Collectively, these findings suggest that practitioners should set goals with athletes which are process or performance based, approach orientated, and with goal specificity tailored to the athlete and the performance context.

Imagery refers to the creation or re-creation of an experience related to the performance execution or outcome [121] and is effective for performing under pressure [4] and overall sports performance [21]. In a meta-study of 29 qualitative studies, Hufton and colleagues [4] reported imagery was often utilised pre-skill execution under pressure and was reported to improve self-confidence and perceived control. A systematic review and meta-analysis [21] reported that the performance benefits of imagery were greater the more an athlete practiced imagery. Athletes, however, may also vary in their ability to generate and control images [122]. For example, exploring elite golfers’ experiences of performance under pressure, Hill and colleagues [123] reported that some athletes lost control of their imagery at critical moments, which contributed to choking. Indeed, it has been suggested that for athletes with low imagery ability, which may be assessed via several methods (see [124]), focus should be placed on supporting athletes to develop their imagery ability (see [125, 126] for development strategies) rather than solely emphasising quantity of practice. As such, considering an athlete's underlying imagery ability and ensuring both adequate and tailored practice can support the effective use of imagery.

Self-talk refers to verbalisations or statements directed towards the self that aim to support performance by the activation of appropriate responses [127, 128]. Self-talk may be goal directed (i.e. deliberately employed to support task performance) or spontaneous (i.e. unintended yet related to the task) [129]. A systematic review and meta-analysis demonstrated self-talk as broadly effective for sport performance [22], whilst a systematic review and meta-study showed self-talk to support performance under pressure [4]. Generally, it has been suggested that motivational self-talk (i.e. reinforcing oneself to achieve the desired outcome) is more appropriate for competitive, pressurised settings, as opposed to instructional self-talk (i.e. clear self-instructions to support skill execution), which may be more appropriate in training [127, 128]. It has also been suggested, however, that instructional self-talk may be more appropriate for fine motor tasks [127]. With these points in mind, the best type of self-talk for athletes may ultimately depend on individual preferences and the sporting context.

Arousal management strategies typically involve the deliberate modification of breathing rate or muscular tension to regulate (and often reduce) arousal. Mesagno and Mullane-Grant [1] reported that use of diaphragmatic deep breathing as a PPR resulted in improved performance under pressure compared with a control group. A systematic review suggested that whilst positive effects have been demonstrated for the effects of acute relaxation interventions on general sport performance, the results remain inconsistent [14]. Meanwhile, a systematic review conducted by Laborde and colleagues [15] reported that longer-term interventions using slow-paced breathing and breath-holding had positive effects on physical performance. Relaxation techniques, however, may not be appropriate for all athletes, such as those aiming to increase arousal prior to performance [16]. Arousal management strategies, therefore, should be context specific.

### Mindfulness-Based Approaches

Mindfulness refers to ‘paying attention on purpose, in the present moment, and non-judgmentally’ [130]. Typically, mindfulness is utilised to support an athlete's ability to remain focused on the environmental cues and demands of task performance, despite any internal discomfort they may experience [131]. Several randomised controlled trials examining performance in naturalistic settings have reported positive effects of various mindfulness-based training programs on perceived training performance [132], coach-evaluated performance [131] and self-evaluated physical performance and emotional regulation [29]. In a recent systematic review and meta-analysis of randomised controlled trials using the Mindfulness Acceptance Commitment protocol, Ptáček et al. [17] reported overall positive effects on performance; however, studies included in this review largely used indirect measures of performance, and mixed results were reported. Similarly, a systematic review of mindfulness and acceptance approaches [19] reported that whilst some studies reported large effect sizes on increasing performance and reducing performance anxiety, the evidence base was inconsistent, with a need for higher quality studies. A systematic review by Bühlmayer et al. [18] demonstrated that mindfulness interventions (i.e. not necessarily comprising acceptance and commitment components) demonstrated positive effects on performance. As such, whilst methodological criticisms exist [e.g. [17, 19], present evidence suggests that mindfulness-based approaches offer a strategy that may be applicable across a range of contexts and sports.

### Summary

A summary of the evidence for the reviewed strategies, including those specific to the training environment, is presented in Table 2. Specifically, this table summarises evidence from studies that have explicitly manipulated or explored pressure, as well as systematic reviews and meta-analyses which have examined the overall performance effects of these strategies (i.e. pressure was not examined as a specific moderator or mediator). The inclusion of this latter evidence is beneficial as it provides further insight into how practitioners can most effectively support athletes’ utilisation of these strategies. Table 2 is not intended to provide a systematic overview of all research on these strategies, but rather, presents an overview of key studies informing the evidence base for the utility of these strategies. Whilst this body of research is extensive, it is important to recognise that there have also been calls for increased methodological rigour in both original empirical research (e.g. [19]) and systematic and narrative review methodologies regarding these strategies [12] (see Recommendation 8, Fig. 2).

**Table 2:** Descriptive summary of evidence for effectiveness of strategies under pressure and general performance

Strategy categorisation	Descriptive summary of evidence for effectiveness of strategies to support performance	Study design and sample
Pressure training		
	Performance under pressure: Moderate effect of pressure training on various performance domains (sport, *k* = 10; law enforcement, *k* = 4), with a moderate effect for sports performance [5]	[5] Systematic review and meta-analysis (*k* = 14)
Implicit learning		
	Performance under pressure: Medium effect of implicit learning on performance under pressure within sports-based tasks, primarily consisting of novice athletes [7]	[7] Systematic review and meta-analysis (*k* = 10)
Pre-performance routines		
	Performance under pressure: Moderate-large positive effects of PPRs on performance in experimental conditions both under low-pressure and pressure conditions. No difference reported in effectiveness between PPRs that were individualised or standardised [8]	[8] Systematic review and meta-analysis (*k* = 33)
Implicit attentional strategies		
	Performance under pressure: Support performance under pressurised conditions and has been demonstrated across different experimental contexts such as golf-putting (dual-task [112], quiet eye training [133]), soccer penalties (quiet eye training [114]), gymnastics (hemispheric priming [113]) and basketball free-throws (dual-task [111])	Experimental ([112] *n* = 20 [133] *n* = 14 [114] *n* = 20 [113] *n* = 21) [111] multiple single case (*n* = 3)
Psychological skills training		
Goal setting	Performance under pressure: Specific goals may support increased motivation and attentional focus under pressure [49, 134]; nonspecific goals may be useful to reduce perceptions of pressure or anxiety [120]. Process goals support task-focused attention [4]	Qualitative ([49] *n* = 26 [134] *n* = 16 [120] *n* = 17) [4] Systematic review and meta-study (*k* = 29)
	Overall performance: Goal setting has a medium positive effect on performance; process and performance goal types provide the greatest performance and psychological benefits [13]. Setting approach-orientated goals has a medium-large positive effect on performance [20]	[13] Systematic review and meta-analysis (*k* = 27) [20] Systematic review and meta-analysis (*k* = 17)
Imagery	Performance under pressure: Imagery utilised prior to skill execution reported to support facilitative interpretations of anxiety, increase self-confidence, improve perceived control and prevent choking under pressure [4]	[4] Systematic review and meta-study (*k* = 29)
	Overall performance: Imagery has a moderate effect on supporting performance. Imagery is dosage specific and is more effective when more sessions are employed [21]	[21] Systematic review and meta-analysis (*k* = 55)
Self-talk	Performance under pressure: Positive and task-orientated self-talk reported to support performance under pressure [4]. Motivational self-talk is most appropriate for competitive settings; however, this may depend on the type of task [128]	[4] Systematic review and meta-study (*k* = 29) [128] randomised controlled trial (*n* = 41)
	Overall performance: Self-talk has a moderate effect on supporting performance, with greater effects seen for fine motor tasks, novel tasks, and when participants were taught how to utilise self-talk [22]	[22] Systematic review and meta-analysis (*k* = 32)
Arousal management	Performance under pressure: Deep breathing as part of a PPR improved performance under pressure compared with control group [1]	[1] Experimental (*n* = 60)
	Overall performance: Relaxation techniques have mixed evidence on performance; 13 out of 21 randomised controlled trials demonstrated a positive effect of relaxation on acute performance [14]. Long-term slow-paced breathing and breath-holding interventions demonstrated a large and small positive effect on performance, respectively [15]. Preparatory arousal strategies have been positively associated with muscular strength, particularly increased muscular power and endurance; however, this effect has been demonstrated in novice rather than trained individuals [16]	[14] Systematic review (*k* = 21) [15] Systematic review and meta-analysis (*k* = 37) [16] Systematic review (*k* = 53)
Mindfulness-based approaches		
	Performance under pressure: Interventions in naturalistic settings have demonstrated significant positive effects on perceived training performance [132], coach-evaluated performance [131] and self-evaluated physical performance and emotional regulation [29]	RCT ([132] *n* = 69 [131] *n* = 18 [29] *n* = 24)
	Overall performance: Moderate positive effects on performance demonstrated for the mindfulness-acceptance-commitment protocol [17] and other mindfulness-based interventions [18]	Systematic review and meta-analysis ([17] *k* = 1 [18] *k* = 9)

*k* = no. of studies; *n* = no. of participants, *PPR* = pre-performance routine

## Evaluating Performance Under Pressure

**Fig. 1:**
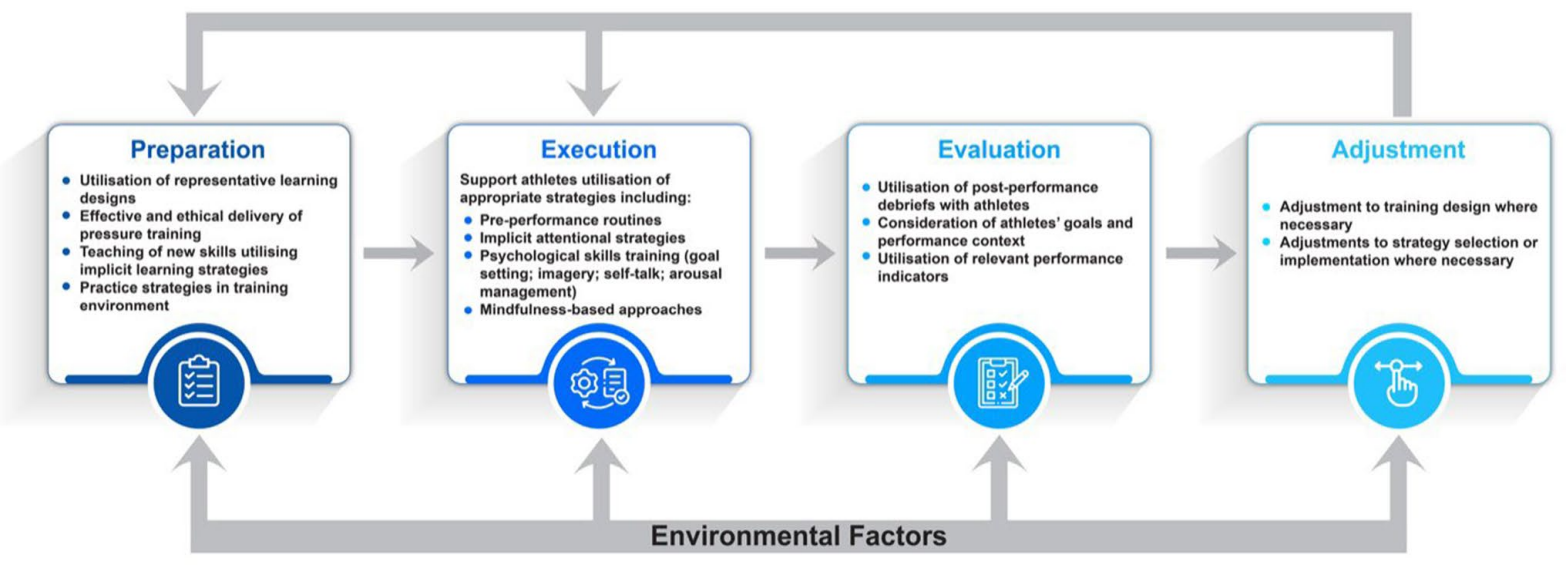
Guiding framework for practitioners to support athlete performance under pressure

**Fig. 2:**
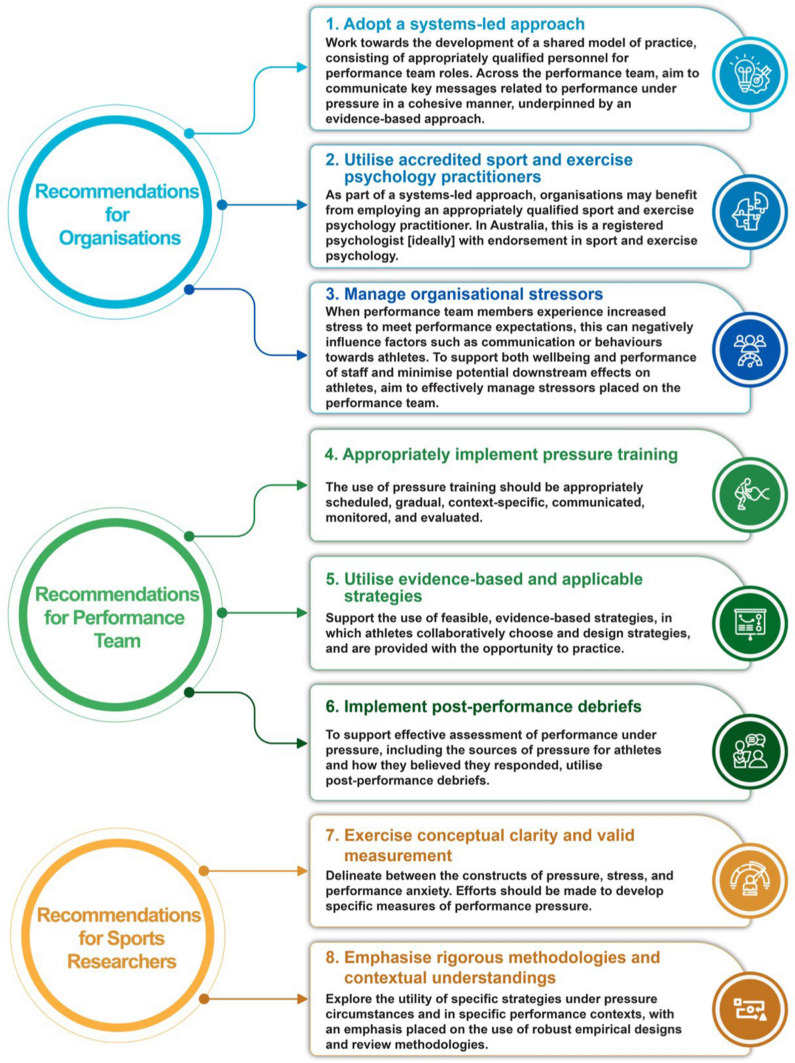
Key recommendations by stakeholder group to support performance under pressure

Post-performance debriefs provide an opportunity to support athletes’ ongoing development and self-awareness whilst offering the performance team information to assess current progress and adjust support ([135]; see Recommendation 6, Fig. 2). Drawing on Chow and Luzzeri [136], we outline several key considerations when conducting post-performance debriefs following performing under pressure. Context plays an important role when evaluating performance under pressure. For example, athletes have reported that performance under pressure is often assessed against their goals, which may be influenced by factors such as their role in the team, the opponent, or the game situation [46, 82]. Given the internal nature of these reflections, there is benefit in considering the athlete's own perceived performance [136]. Coaches often play an important role in setting such goals for athletes [82] and there may be further benefit in also considering the coach's perception of the athlete's performance under pressure (e.g. [137]). Assessing the athlete's experience of pressure, and how this may have impacted their performance, may comprise part of the post-performance debrief. Given the limitations regarding psychometric measures of pressure (e.g. [48]; see Recommendation 7, Fig. 2), qualitative discussions of the athlete's experience may be most practical. In addition, physiological measurements may offer complementary insights into the experience of pressure, stress, or performance anxiety. For example, anticipatory cortisol responses have been identified in athletes prior to competition (however, this may differ based on gender and level of expertise; see [138]), and whilst evidence is mixed (see [68]), it has been proposed that cardiovascular reactivity patterns are suggestive of athletes experiencing challenge or threat states [119]. Lastly, debriefing on the athletes’ use of strategies to support their performance under pressure, and the perceived implementation of these, can provide a platform for necessary adjustments to both individual strategies and the broader training environment. Figure 1 provides a descriptive overview of the process to support athletes’ performance under pressure.

## Conclusions and Recommendations

Performing under pressure is an essential component of sports performance. The aim of this position statement was to provide sports practitioners with a synthesis of scientific literature regarding the sources of pressure athletes may experience, how athletes may respond to this pressure, the different strategies that may be used to support performance under pressure, and considerations for evaluating performance under pressure. Recommendations for organisations and performance team members to guide their practice when working with athletes who perform under pressure, and for sports researchers to continue the development and generation of robust knowledge regarding performance under pressure, are provided in Fig. 2.
